# Mechanical metamaterials and *beyond*

**DOI:** 10.1038/s41467-023-41679-8

**Published:** 2023-09-26

**Authors:** Pengcheng Jiao, Jochen Mueller, Jordan R. Raney, Xiaoyu (Rayne) Zheng, Amir H. Alavi

**Affiliations:** 1 Ocean College, Zhejiang University, Zhoushan, Zhejiang China; 2 Department of Civil and Systems Engineering, Johns Hopkins University, Baltimore, MD USA; 3 Department of Mechanical Engineering and Applied Mechanics, University of Pennsylvania, Philadelphia, PA USA; 4 Department of Materials Science and Engineering, University of California, Berkeley, CA USA; 5 Department of Civil and Environmental Engineering, University of Pittsburgh, Pittsburgh, PA USA; 6 Department of Bioengineering, University of Pittsburgh, Pittsburgh, PA USA

**Keywords:** Materials science, Engineering

## Abstract

Mechanical metamaterials enable the creation of structural materials with unprecedented mechanical properties. However, thus far, research on mechanical metamaterials has focused on passive mechanical metamaterials and the tunability of their mechanical properties. Deep integration of multifunctionality, sensing, electrical actuation, information processing, and advancing data-driven designs are grand challenges in the mechanical metamaterials community that could lead to truly intelligent mechanical metamaterials. In this perspective, we provide an overview of mechanical metamaterials within and beyond their classical mechanical functionalities. We discuss various aspects of data-driven approaches for inverse design and optimization of multifunctional mechanical metamaterials. Our aim is to provide new roadmaps for design and discovery of next-generation active and responsive mechanical metamaterials that can interact with the surrounding environment and adapt to various conditions while inheriting all outstanding mechanical features of classical mechanical metamaterials. Next, we deliberate the emerging mechanical metamaterials with specific functionalities to design informative and scientific intelligent devices. We highlight open challenges ahead of mechanical metamaterial systems at the component and integration levels and their transition into the domain of application beyond their mechanical capabilities.

## Abstract (web summary)

Mechanical metamaterials are known for their unconventional mechanical properties. In this perspective, the authors give an overview of the current state of mechanical materials research and suggest a roadmap for next-generation active and responsive mechanical metamaterials.

## Introduction

**Fig. 1:**
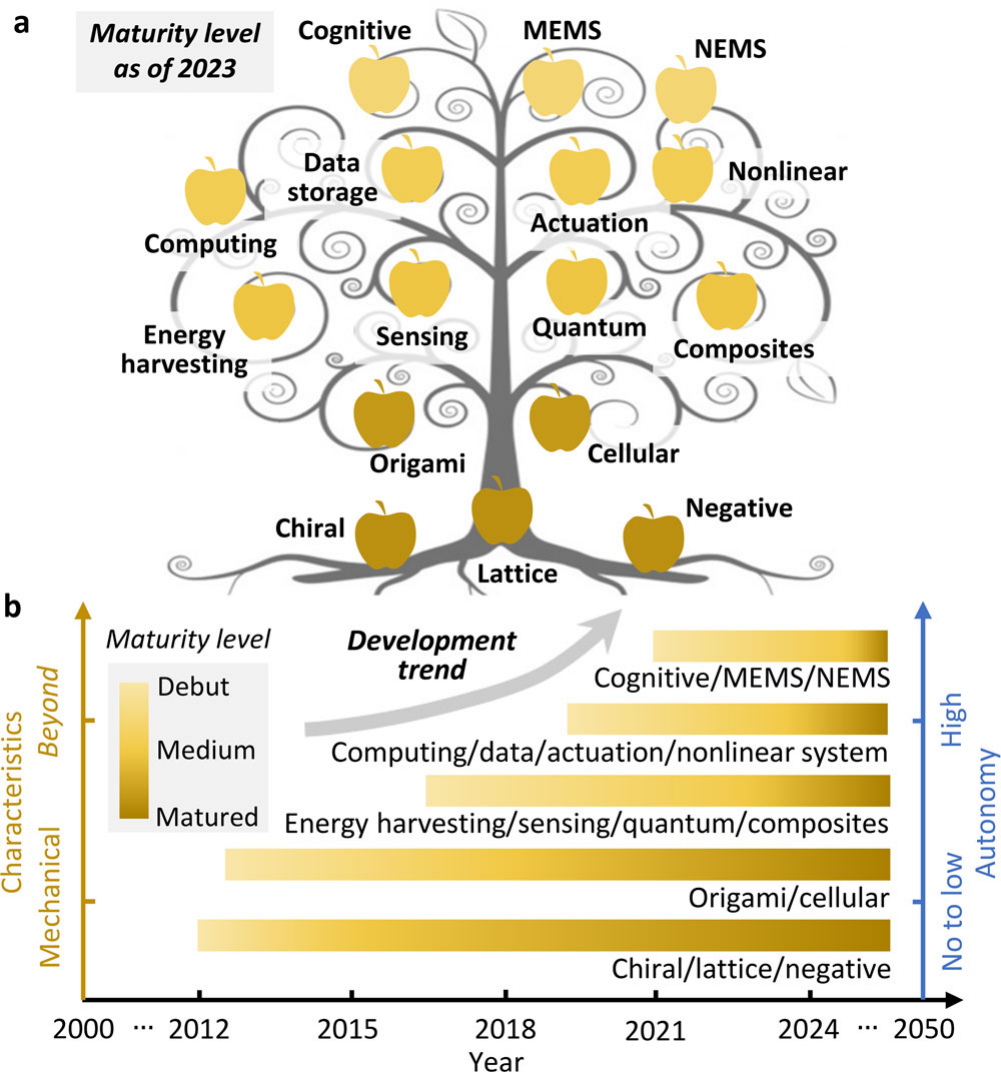
Mechanical metamaterial tree of knowledge. **a** Progression and future of mechanical metamaterials within and beyond the mechanical functionalities and toward achieving a level of cognition and autonomy. **b** Maturity level of mechanical metamaterials with the development trend leading to cognitive integrated mechanical metamaterial systems.

Mechanical metamaterials can achieve distinct and exotic mechanical properties through the rational design of their microstructures^1–4^. Obtaining programmable behavior through the interplay between material and structure in mechanical metamaterials enables integrating advanced functionalities into their texture beyond their mechanical properties. Examples are mechanical metamaterials capable of sensing^5–7^, energy harvesting^8–12^, actuation^13–15^, adaptation^16^, computation^17,18^, information processing^19,20^, among others. Mechanical metamaterials have shown their potential as building blocks for multifunctional intelligent matter^21–23^. Yet, researchers have only begun to scratch the surface of what could be an immense scientific field. Figure 1a illustrates the “mechanical metamaterial tree of knowledge” within and beyond the mechanical domain, categorizing mechanical metamaterials with respect to *multifunctionality* and *autonomy*. The tree is inspired by several studies with focus on the future direction of the entire metamaterials family, including the well-established optical and electromagnetic metamaterials (e.g^24–33^.). Figure 1b illustrates the maturity level of typical mechanical metamaterials within and beyond the mechanical domain and outlooks the development trend that leads to integrating a level of artificial cognition into the mechanical metamaterial fabric. The tree implies that chiral, lattice and negative metamaterials (e.g., negative bulk modulus or negative elastic modulus) are ripe followed by origami and cellular metamaterials^27–29^. Recent research trends have been entering a space beyond merely exploring unprecedented mechanical properties. Emerging directions envisioned are sensing^30,31^, energy harvesting^32,33^, and actuating^34^ mechanical metamaterials. Based on the dynamic features of architected, photonic metamaterials^35,36^, topological wave physics has also been reported as a promising direction for mechanical metamaterials in recent studies^37–40^.

Despite its capacity to generate new generations of mechanical metamaterials, the entire concept of composite mechanical metamaterials is still in its infancy. However, the tree of knowledge reveals that digital computing, digital data storage, and micro/nano-electromechanical systems (MEMS/NEMS) applications are one of the pillars of the mechanical metamaterials future research. The tree also shows the road to achieve full autonomy for mechanical metamaterials. Along this direction of evolution, the final target can be mechanical metamaterials with a level of cognition. Cognitive abilities are crucial elements in a truly intelligent mechanical metamaterial. Cognition and intelligence are intertwined but distinct concepts. Cognition is a process through which knowledge is acquired, organized, and processed. Intelligence integrates cognitive abilities for learning from experiences, comprehending complex situations, adapting, and responding purposefully. Similar to complex living organisms, intelligent mechanical metamaterials can potentially deploy their cognitive abilities for sensing, self-powering, and information processing to interact with the surrounding environments, optimizing their response, and creating a sense–decide–respond loop. Intelligent mechanical metamaterial can realize these advanced functionalities through the rational design of their structures using responsive materials or living biological cells. Interestingly, such multifunctional materials systems already inherit all outstanding mechanical features of classical mechanical metamaterials enabling them to operate and survive in various environmental conditions.

This perspective article explains various research domains related to the mechanical metamaterial tree of knowledge. The overarching aim is to provide new roadmaps for the design and discovery of mechanical metamaterials with advanced functionalities. We highlight what unprecedented/counterintuitive mechanical characteristics can be achieved by mechanical metamaterials and how; what advanced functionalities are expected to be achieved by mechanical metamaterials beyond the mechanical domain, how they can enable creating multifunctional intelligent matter; how to surpass the challenges of mechanical metamaterials at the component and integration levels, and when mechanical metamaterial devices and systems with a level of cognitive capabilities and intelligence are expected to be applied beyond the mechanical domain. Starting with a review of mechanical metamaterials developments in all domains (Box 1), we explain their advantages and limitations with respect to multifunctionality and responsiveness, adaptability, actuation, and autonomy. We then discuss data-driven and, in particular, artificial intelligence (AI) methods for inverse design and optimization of mechanical metamaterials. We present major advances in mechanical metamaterials which can lead to the invention of informative and computing devices. The remainder of this perspective article is organized as follows. Section 2 summarizes the main characteristics of mechanical metamaterials within the mechanical domain. Section 3 provides an overview of the current capabilities of mechanical metamaterials beyond their mechanical properties. Section 4 outlines a vision for future mechanical metamaterials devices. Section 5 summarizes the main conclusions in this perspective article.

Box 1 Mechanical metamaterials in the materials history *from* natural to manmade materials The main development history in materials science is the history from natural to manmade materials. As a type of manmade structural materials assembled by microstructures, mechanical metamaterials have started attracting remarkable research attention since 2010s.

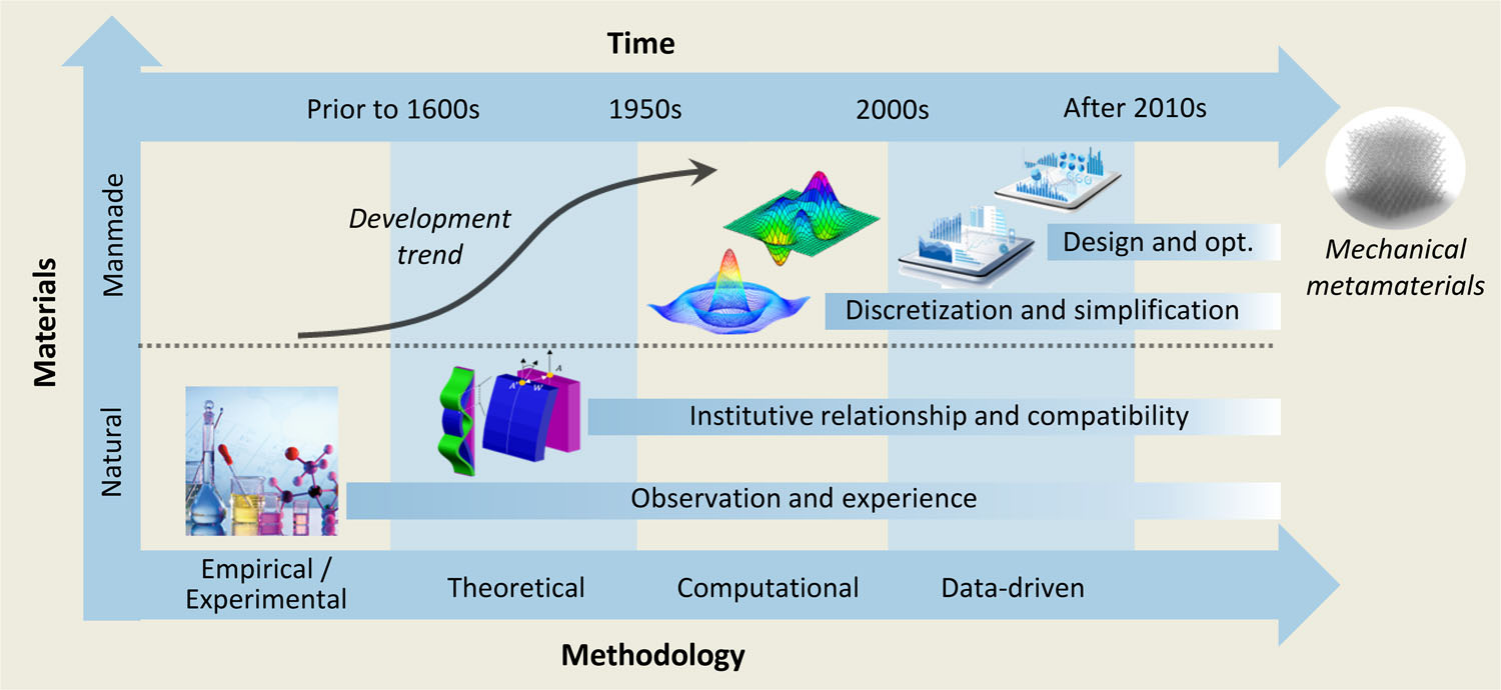

### Debut, recent developments, and current situation

**Fig. 2:**
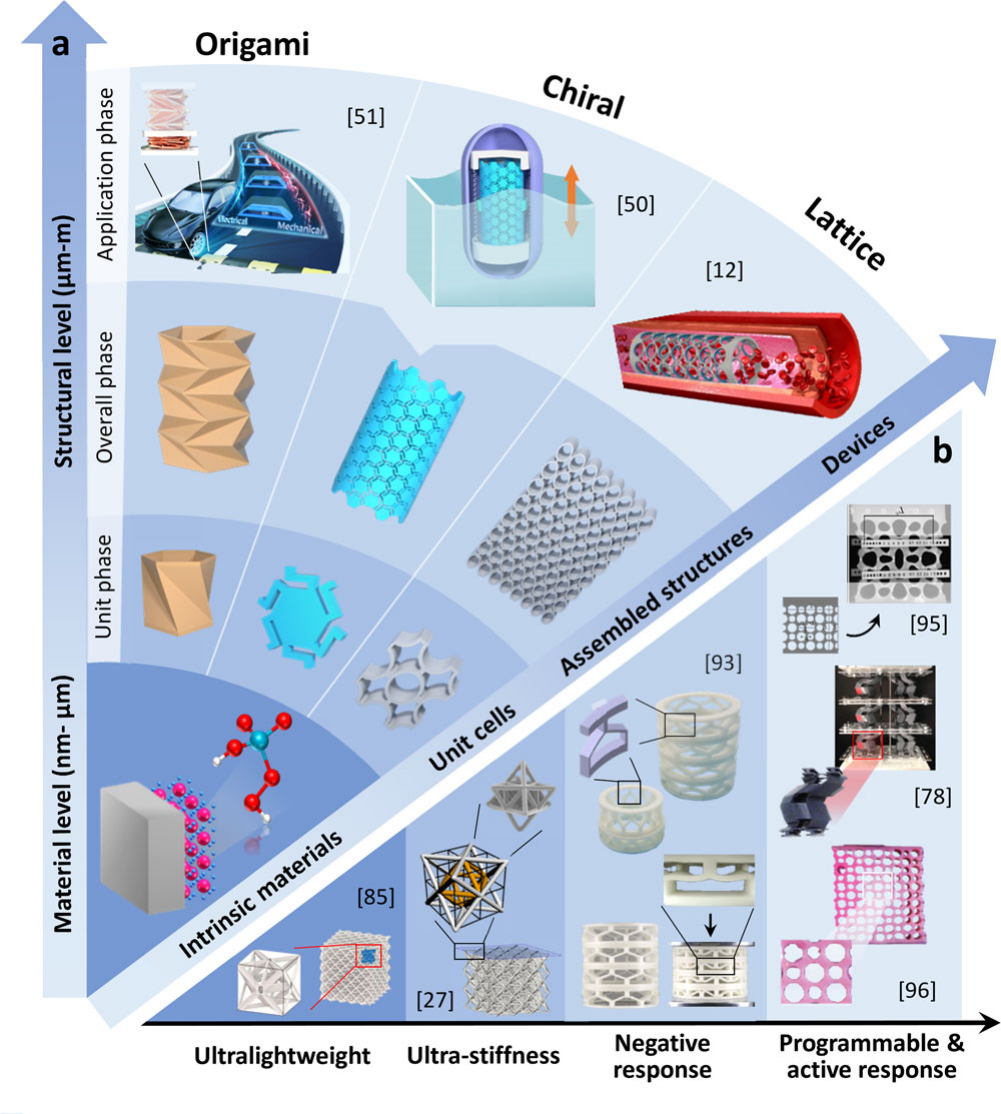
Principles, main categories, and properties of mechanical metamaterials. **a** Formation of mechanical metamaterials at the material to structural levels. Categories of mechanical metamaterials, such as origami, chiral, and lattice metamaterials, based on the microstructures and their typical applications^12, 50, 51^. **b** Extraordinary mechanical characteristics of mechanical metamaterials as ultra-lightweight, ultra-stiffness, negative response, and programmable response^27, 78, 85, 93, 95, 96^.

The progress of human civilization has been extensively related to the development of tools, which is significantly affected by the discovery and application of materials such as metals, wood, textiles, etc. Properties and characteristics of these materials dominate the functionality of these tools, and thus, it is desirable to create manmade materials with predominant, controllable material properties^41–44^. To this end, mechanical metamaterials have been proposed as a type of artificial materials with rationally-designed microstructural units to achieve unprecedented mechanical properties^45–49^. Figure 2a illustrates the formation of mechanical metamaterials from the material and structural levels^12,50,51^. The *nm* to *µm* material level determines the material properties. The unit phases for the structural level and application phases range from *µm* to *mm* and the *mm* to *m*, respectively. The unit phase at the structural level refers to the microstructural unit cells. Composition of the periodic unit cells to form mechanical metamaterials is determined by the overall phase. The application phase functionalizes mechanical metamaterials to devices for various applications^52^. The unit cells in the unit phase can be similar while the assembled structures in the overall phase are relatively different, which leads to the overlap between the category of mechanical metamaterials. For example, origami and lattice cells share similar structural characteristics; however, origami and lattice metamaterials are typically grouped in separate categories due to the folding nature of the former and periodic assembly nature of the latter^2,53^. In the unit phase, the microstructures offer a localized structure-like performance. In the overall phase, the performance resembles homogenous materials^53^. Therefore, mechanical metamaterials may be classified as the type of architected materials whose performance is between the natural materials dominated by their intrinsic material properties and the man-made structures influenced by their structural characteristics^54^. Given the significant dependence of mechanical metamaterials on their representative unit cells, their tunability is typically achieved by rational tailoring of their unit cells. This leads to the possibility of obtaining configurations with desirable mechanical properties^55–58^. While the material level of mechanical metamaterials tends to focus on the mechanism of intrinsic materials, the structural level pays more attention to performance and applications^50,59–62^. Hence, performance needs and application requirements of mechanical metamaterials are typically satisfied by the design and optimization of their microstructural units^63–66^. A major research gap is at the material level where new characteristics should be explored for mechanical metamaterials by incorporating functional materials into their composition^34,67,68^.

Mechanical metamaterials reported in the literature are mainly studied at the structural level. In particular, mechanical metamaterials are obtained by designing different microstructural units in certain assembly manner in the unit phase, and presented by different overall performance (e.g., negative Poisson’s ratio^69–71^) and responses (e.g., negative stiffness^72–74^) in the overall phase. Mechanical metamaterials can be classified according to the design of the microstructural units in the unit phase, for example, as origami, chiral, and lattice metamaterials (Fig. 2a). Origami metamaterials are the 3D structures obtained by folding 2D sheets following certain periodic patterns and shapes^75,76^. Origami metamaterials are designed using origami microstructures^77,78^. Origami metamaterials have been reported to have desirable mechanical characteristics controlled by the order, number, and orientation of folds^79,80^. In order to build chiral metamaterials, left- or right-handed unit cells should be used. These unit cells cannot be superimposed on the mirror images^81^. Chiral metamaterials are typically created using periodic polygons with chiral ligament connections^82–84^. Lattice metamaterials are periodically assembled by numerous uniform lattice cells (e.g., nodes and struts), which are originally inspired by natural lattice solids such as foams^85,86^. Lattice metamaterials are composed of multiple tessellated lattice elements^87^. As a typical kind of lattice metamaterial, cellular metamaterials are obtained using origami design strategy in lattice metamaterials^88,89^. Designing micro/nanostructures in new patterns, cellular metamaterials are often 3D structures composed of interweave tessellations or individual origami layers stacked into lattice patterns^90–92^.

Some of the well-studied mechanical characteristics of mechanical metamaterials include ultra-lightweight, ultra-stiffness, negative response (e.g., negative Poisson’s ratio, negative stiffness) and programmable response, as shown in Fig. 2b^27,78,85,93–96^. Ultra-stiffness was initially reported as the deformation stiffness of mechanical metamaterials in the loading direction^97–100^. Ultra-lightweight is another key mechanical characteristic since ideal construction materials are expected to be ultra-stiff and ultra-light^101^. However, it is very challenging to simultaneously tune the stiffness and density of materials. Mechanical metamaterials enable tackling this issue and designing material systems with tunable stiffness and density^102^. For instance, ceramic octet-truss nanolattices have been reported, with beam thickness of ~5 nm, density of ~6.3 kg/m^3^, and a scaling relationship between the Young’s modulus (*E*) and relative density (*q*) of *E~q*^1.76^^85^. Another study found a scaling relationship of *E~q*^1.61^ for ceramic octet-truss nanolattices with beam thickness of ~60 nm and density of ~258 kg/m^3^^103^. Negative stiffness means that the metamaterial deforms in the same direction as the external force to assist with the deformation^104,105^. Negative stiffness often results in extreme deformation, associated with instability. An example of negative Poisson’s ratio is when a material undergoes negative volumetric changes under compression^29,45^. Mechanical metamaterials with negative Poisson’s ratio can be categorized into two major categories^56,106^, including the negative response that is obtained because of the cellular structure of mechanical metamaterials or the combination of different materials. Table 1 presents the main mechanical categories and typical mechanical design goals for mechanical metamaterials.

**Table 1:** Main mechanical categories of mechanical metamaterials and their typical mechanical characteristics

Formation			
Material level		Material	
		Metallic	Polymeric
Mechanism	Constitutive relationship	• Work hardening constitutive relationship (e.g., Johnson-Cook model and Zerilli-Armstrong model) • Dynamic recovery constitutive relationship (e.g., Arrhenius model) • Dynamic recrystallization constitutive relationship (e.g., Sellars model) • Unified constitutive relationship (e.g., Miller model and Walker model)	• Thermoviscoelasticity-based models • Phase evolution-based models
**Structural level**		**Overall**	
		**2D beam or plate**	**3D cube and others**
	Origami or kirigami	• Origami metamaterials with square twist^76^ • Reconfigurable origami metamaterials^81^	• 3D transformable origami metamaterials with multiple degrees of freedom^79^ • Reentrant origami metamaterials^71^ • Programmable self-locking origami metamaterials^78^
Unit	Chiral	• Chiral, anti-chiral and hierarchical honeycombs^59,66^ • Double-negative mechanical metamaterials^73,76^	• 3D chiral metamaterials with a twist^48^ • Self-rotating 3D chiral mechanical metamaterials^83^ • 3D chiral metamaterials with topological design^66^ • 3D chiral metamaterials with modular design^57^
	Lattice	• Hierarchical lattice materials^86^ • Functionally graded cellular composites with auxetics^150^ • Cellular flexible metamaterials^172^	• Nanolattices^1,85,173^ • Alternating pentamode lattices^87,174^ • 3D plate-lattices^54^ • Reversibly assembled cellular composite materials^88^ • 3D cellular metamaterials with anti-chiral topology^91^

Performance			
Mechanical characteristics		Current status	
		Advantages	Limitations
Response	Ultra-stiffness	• Impact resistance^2,99^ • Energy absorption • Vibration reduction	• Difficulties in design, characterization and application^175^ • Difficulties in fabrication (e.g., ultra-fine complex nanostructures, multi-material systems and super-large structures)^176^
	Ultra-lightweight	• Sound insulation, absorption and reduction^54,104^ • Low consumables and cost	
	Negative response	• Negative Poisson’s ratio (e.g., shear, impact and damage resistance, and energy absorption)^45,69,71,177^ • Negative Stiffness (e.g., large bearing capacity and small deformation, and low natural frequency)^73^ • Negative thermal expansion (e.g., high thermal and electrical conductivity)^178,179^	
	Programmable response	• Controllability • Tunable stimuli	

### Mechanical metamaterials beyond mechanical properties

Recent research efforts have begun to explore the advanced performance of mechanical metamaterials beyond mechanical characteristics. Although mechanical metamaterials are primarily defined by structure-induced mechanical superiority, it is desirable to expand such superiority to other fields by manipulating their material level features through using various types of functional materials. Examples are found in thermal materials;^34,107–109^ energy harvesting^110–113^, power absorption^114,115^, energy storage^116,117^ and monitoring;^118,119^ magnetic materials for electromagnetic energy harvesting^8^ and absorption^120–122^, etc. Table 2 summarizes the mechanical metamaterials beyond mechanical and their main functionalities. Some of the emerging functionalities and applications are based on similar electromechanical, thermomechanical, magneto-mechanical or optomechanical principles.

**Table 2:** A summary of the characteristics of mechanical metamaterials beyond mechanical properties and their main functionalities

Programmability				
	Functionality beyond mechanical properties			
	Energy harvesting and sensing	Soft robotics	Scientific computation	Integrated system
Actuation	• Tactile sensors^5^ • magnetic-based transduction method^5^ • Multiple sensitivity regimes^5^ • High design degrees of freedom^5^	• Programmable actuation of metastructures^103^ • Artificial muscle^103^ • Temperature-dependent switching and information encryption^103^	• A tileable mechanical metamaterial with stable memory at the unit-cell level^128^ • Magnetic actuation^128^ • Reprogrammability^128^	• Origami-inspired 3D programmable metamaterials^79^ • Actuating of unit cells^79^ • Deployable space structures^79^ • SMP-hydrogel stent to deliver drugs^131^
Responsiveness	• Easy-to-manufacture soft tactile sensor^5^ • 3D printing^5^ • Multiple desired sensitivities^5^	• Electro-responsive active metamaterials (e.g., SMPs, SMAs, dielectric elastomers of electroactive polymers, etc.)^131^ • Magneto-responsive active metamaterials^131^ • Light-responsive active metamaterials^131^	• A bi-stable logic-gate elastic metamaterial^22^ • Wave logic operations^22^ • Active regulation without continuous-consuming energy^22^	• Dynamic smart shape transformation^32^ • Thermal or photothermal trigger^32^ • Mechanical actuators^32^
Adaptability	• Multi-layer structure; Multiple perfect absorption bands^180^ • Solar energy harvesting^180^	• Reconfigurable structures composed of multistable unit cells^139^ • 2D graded structures exhibiting serpentine motion^139^	• Cellular mechanical metamaterials composed of conductive polymers^181^ • Digital logic gates and gate assemblies^181^	• Adaptive mechanical properties^181^ • 4D printing^181^ • LED integrated device^181^ • Biomedical scaffold^181^
Autonomy	• Molecular self-assembly of block copolymers (BCPs)^182^	• Origami-inspired structures^80^ • Mechanical self-folding technique^80^ • Microrobots^80^	• Reprogrammable mechanological metamaterials^129^ • Electromagnetic excitation^129^ • Mechanical systems with embedded intelligence^129^	• Mechanical adjustable medical stent^183^ • Radial expansion ability enhancing^183^

Responsiveness				
	Applications beyond mechanical properties			
	Energy harvesting and sensing	Soft robotics	Scientific computation	Integrated system
Magnetic	• Multistable mechanical metamaterials composed of multiple magnets systems^65^ • Energy trapping^65^	• Cilia-inspired soft robots^144^ • Programmable magnetization patterns^144^ • Magnetic actuation^144^ • Magneto-mechanical metamaterials^135^ • Shape and property tunability^135^ • Coupled magneto-mechanical actuation^135^	• A tileable mechanical metamaterial with stable memory at the unit-cell level^128^ • Magnetic actuation^128^ • Reprogrammability^128^	• Cellular metamaterials^184^ • Magneto-elastic coupling^184^ • Omni-directional, multi-modal energy absorption in a low-density, tunable, and re-usable platform^184^ • Magnetic field responsive mechanical metamaterials^185^ • Dynamic control and on-the-fly tunability^185^
Electrical	• Kirigami/origami structures^59,61^ • Triboelectric nanogenerators (TENG)^59,61^ • Self-aware composite mechanical metamaterials^12^ • Self-powering and self-sensing blood vessel stents and shock absorbers^12^	• Electro-responsive active metamaterials (e.g., SMPs, SMAs, dielectric elastomers of electroactive polymers, etc.)^131^ • Robot fabric^131^ • Micrometer-scale origami quadruped robot^131^ • Metamaterial enabled high-speed micro-robot with self-sensing^186^	• Conductive polymers^23^ • Digital logic gates and gate assemblies^23^	• Frequency coding metamaterials^121^ • Electromagnetic energy radiations control^121^ • Lamellar ferroelectric metamaterials^68^ • Excellent printability^68^ • High piezoelectric behaviors^68^ • Smart biological systems^68^ • Triboelectric nanogenerator-enabled structural elements^60^ • Civil infrastructure monitoring systems^60^
Thermal	• Broadband thermal energy extraction^187^ • Hyperbolic metamaterials^187^ • High near-field thermal energy transfer rate^187^	• Self-triggered thermomechanical metamaterials^15^ • Temperature-induced microgrippers^15^ • Temperature-responsive multistable metamaterials^15^ • Autonomous actuation and adaptation to the environment^15^	• Chemical logic gates^188^ • Standalone systems to analyze different stimuli^188^	• Thermomechanically triggered two-stage pattern switching of 2D lattices^189^
Light-driven	• Thermophotovoltaics energy conversion^107^ • Frequency-selective surface^107^ • Metamaterial emitters^107^ • Broadband and omnidirectional hot electron photodetectors^190^ • Metamaterial perfect absorbers^190^	• Light-responsive film actuators^131^ • 2D-to-3D structural transformations^131^ • Bionic micro-robots^131^	• Photonic computing^191^ • Photo-responsive supramolecular polymers^192^	• Frequency reconfigurable and programmable antennas^131^ • Five more different working modes and programmable frequency reconstruction^191^ • Light-driven soft actuators^192^ • Light-triggered electronic devices^192^

### Programmable response: adaptability, actuation, and autonomy

**Fig. 3:**
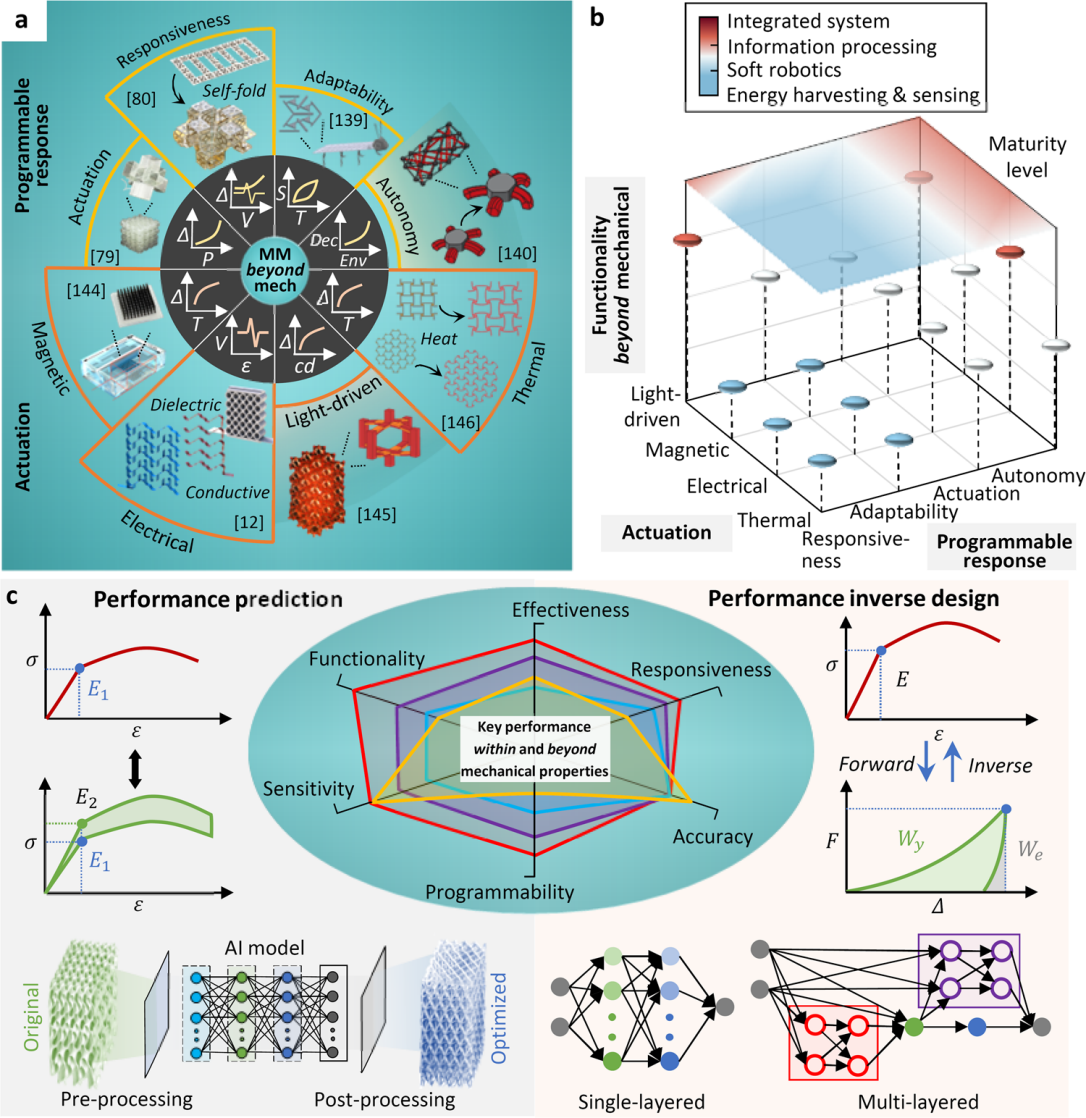
Mechanical metamaterials beyond mechanical properties. **a** Programmable response of mechanical metamaterials in responsiveness, adaptability, actuation and autonomy^79, 80, 139, 140^, and actuation of mechanical metamaterials subjected to electrical, thermal, magnetic and light-driven excitations^12, 144–146^. **b** Maturity levels of the functionalities beyond mechanical in energy harvesting, soft robotics, information processing, and integrated system with respect to programmable response and actuation. **c** AI-enhanced mechanical metamaterials in performance prediction that collects and processes response data to train and validate algorithms to develop AI models to predict key response such as stress-strain relationship, and performance inverse design that inverses the procedures to determine the variables based on predefined response.

Programmable response is an emerging direction for mechanical metamaterials beyond mechanical properties^96,123–127^. Electrical responsiveness is an important functionality for designing adaptive, actuating, and autonomous mechanical metamaterials^128–130^. For example, research ideas have been opened by active and adaptive mechanical metamaterials that design electrical materials into the microstructural units of metamaterials to autonomously convert mechanical-strain input into electrical-signal output^6,12,50^. As a consequence, active and adaptive mechanical metamaterials have been developed with outstanding mechanical properties, electrical performance, and excitation sensitivity^33,131^. Significant work has also been done on energy harvesting from various sources in the environment, such as mechanical waves^31,111^, acoustic sources^132–134^, etc., and sensing and monitoring in different application scenarios such as civil infrastructures^12,60^, vehicle velocity^51^, etc. Self-actuated mechanical metamaterials are obtained by integrating with functional materials such as magnetic-^135,136^, thermal-^34,109,137^, and electrical-driven^31,50,67,138^ materials. Mechanical materials with the ability to adapt, actuate, and exhibit some degree of autonomous behavior are summarized in Fig. 3. These efforts can be built upon by discovering new functional materials and optimizing microstructures at the material level to effectively stimulate these functional materials at the structural level.

Figure 3a displays a summary of programmable mechanical metamaterials reported in the literature^79,80,139,140^. Active tunability and programmability are two major emerging directions for mechanical metamaterials^128,129,141^. In active mechanical metamaterials, time is considered as an additional dimension such that mechanical metamaterials can be dynamically adjusted over time to obtain new functionalities in advanced devices^33,131^. Active mechanical metamaterials are generally created with various functional materials such as shape memory polymers (SMPs)^22^, shape memory materials (SMMs)^13^, etc. Using functional and self-adaptive materials enables realizing active metamaterials that can automatically respond to different external stimuli^33,131,142,143^. Programmable mechanical metamaterials often refer to tunable mechanical characteristics (e.g., stiffness^99,100^, Poisson’s ratio^29,45,47^ and elastic wave propagation^31^), or overall tunable characteristics (e.g., the ability to adapt in response to external excitations^139^ and self-stimulated under certain stimuli^13^). The second type of tunability is achieved using functional materials and is an important element to design active mechanical metamaterials.

### Actuation in response to electricity, heat, magnetic fields, and light

Integrating functional materials and mechanical design is an emerging research area to explore responsive mechanical metamaterials^12,144–146^ (Fig. 3a). Recent studies have revealed the possibility of designing mechanical metamaterials with efficient energy harvesting and electrical performance, i.e., mechanical energy metamaterials^50^. Researchers have studied the electrical response of this class of metamaterials to different excitation types such acoustic^18,111,120^, thermophotovoltaic^107^ and magnetic^120,121^. Hyperbolic^114^, lattice^54^, and multistable microstructures^65^ have been used in energy harvesting and energy absorption applications in mechanical metamaterials, using functional materials (e.g., metallic microlattices^28^) and by different fabrication technologies (e.g., additive manufacturing^29^). Energy harvesting mechanical metamaterials (e.g., piezoelectric^31,67^ and triboelectric^6,7,12,30^) are developed to generate electrical power in response to vibrations^46,62^ and waves^111,117^.

In addition, mechanical metamaterials composed of rationally chosen thermal or photovoltaic materials can serve as thermal energy harvesters^34,107^. Different from triggering mechanical metamaterials by temperature fluctuation for configuration or property changes, thermal mechanical metamaterials have been designed to generate electricity. Implanting or embedding magnetic materials in mechanical metamaterials, magnetic mechanical metamaterials are developed as multistable microdevices^8,120,121^. Memory mechanical metamaterials were developed using the mechanical bits consisting of magnetic-to-mechanical binary elements. The mechanical bits can be switched between bistable states under magnetic actuation. Light-driven materials such as liquid-crystal elastomers have been used to 3D print mechanical metamaterials that are stimulated under external light from a blue LED^145^. Figure 3b compares the maturity levels of the functionalities beyond mechanical properties with respect to the programmable response and actuation. Energy harvesting, sensing, and soft robotics are relatively mature functionalities, followed by basic information processing capabilities. The integrated systems are found with the least maturity level.

### AI-driven inverse design and prediction

Data-driven techniques have been recently used for inverse design of mechanical metamaterials and optimizing their complex microstructures^147–149^. Traditional experimental, theoretical and computational research paradigms have encountered technical bottlenecks in design, analysis and fabrication of mechanical metamaterials due to the vast design space^150,151^. Applications of the data-driven methods, particularly AI-based approaches, in mechanical metamaterials are mainly in the two directions of performance prediction and inverse design, as shown in Fig. 3c. AI is used to describe the complex relationships between inputs (e.g., material and structural level parameters) and outputs (e.g., mechanical characteristics and beyond). AI models have been recently developed to assess the structural properties of mechanical metamaterials^141,152^, such that to address the technical challenges of fabricability in industrialized fabrication^153^, complexity in microstructural validation^154^, designability^155^, and optimization in performance control^156^.

In general, AI can contribute to the mechanical metamaterial science in four areas: finding the trade-off between microstructural complexity and fabrication feasibility, optimization to maximize/minimize certain characteristics, response prediction, and inverse design of microstructures for a designated functionality. The AI methods are capable of exploring functional materials at the material level (e.g., nano-composites) and microstructures at the structural level (e.g., cellular or origami units)^153^. They can clarify the relationship between the microstructures and the characteristics within and beyond mechanical, and predict and optimize the characteristics^154^. They may also be used to develop optimization tools for designing more complex functional metastructures^155,156^.

More recently, inverse design has been reported to obtain mechanical metamaterials with performance-oriented characteristics. Here, performance-oriented implies that a specific performance is predefined as a target to optimize mechanical metamaterials^157,158^. The AI paradigms can be used to process response data obtained from various performance-oriented tests on mechanical metamaterials, train and validate the appropriate algorithms to predict the response of mechanical metamaterials, analyze the sensitivity of inputs, approach the desirable response by tuning the dominant variables, and inverse the procedures to determine the dominant variables of mechanical metamaterials based on predefined responses. To this end, there are challenges to be addressed in the future: (1) establishing robust and comprehensive databases to calibrate the AI models, (2) minimizing the computation costs for extensive simulations often required to explore the mechanical metamaterials design space; (3) developing uncertainty quantification approach for the AI models, and (4) introducing physics-based approaches and physical constraints to improve the reliability and performance of the AI models^155^. Table 3 compares the AI algorithms used for the prediction of mechanical metamaterials properties and their inverse design in recent studies.

**Table 3:** The AI algorithms used for the prediction of mechanical metamaterials properties and their inverse design

AI algorithms	Advantages	Disadvantages	Prediction		Inverse design	
			Type	Ref.	Type	Ref.
Artificial neural networks	• Flexibility and scalability • Computationally efficient • Parallel processing • Powerful ability to extract features in data	• Accuracy relies on amount of data • Prone to overfitting • Black box nature	Nonlinear mechanical metamaterials and fractal metamaterials	^157,193^	Inflatable soft membranes	^158^
Deep learning^a^	• Powerful ability to extract features in data • Handling complex Data • Parallel processing • Flexibility and scalability	• Computationally intensive • Accuracy relies on large amount of image data • Prone to overfitting • Black box nature	Copper spheres embedded in polylactide matrices	^194^	2D and elastic mechanical metamaterials	^153,195^
			Tetra-chiral auxetics, cellular metamaterials	^196,197^	Gradient mechanical metamaterials	^155,198^
			Magneto-mechanical metamaterials and auxetic kirigami metamaterials	^199,200^	Metasurfaces, magneto-mechanical metamaterials and auxetic mechanical metamaterials	^154,199,201^
Evolutionary strategy^b^	• Exceling at global optimization • Scalability and invariance • Built-in feature selection • High interpretability • Robustness to noise • Less susceptible to overfitting	• Computationally intensive • Fixed standard deviation parameter of noise • Slow search speed	Nanoscale corrugated plates	^202^	2D and 3D mechanical metamaterial with nonlinear response, fractal metamaterials	^157,193,203^
Genetic programming	• Global search ability • Scalability • Simple process • Built-in feature selection • High interpretability • Robustness to noise	• Computationally intensive • Complicated programming implementation • Slow search speed	Graphene origami metamaterials	^204,205^	Auxetic mechanical metamaterials with zero Poisson’s ratio	^206^
Bayesian network classifiers	• High learning efficiency • Small time and space overhead in classification	• Computational complexity • Dimensional challenges in computing probability • Low interpretability	--	--	Mechanical metamaterials with negative stiffness	^151^
Decision trees	• Simple data preparation • High interpretability • High efficiency and accuracy	• Prone to overfitting • Bias toward features with more levels • Difficulty in handling missing data	Non-rigid square-twist origami	^207^	--	--

^a^Deep learning is a specialization of artificial neural networks with multiple hidden layers.

^b^Evolutionary strategy and genetic programming are branches of evolutionary computation.

### Emerging mechanical metamaterial devices

**Fig. 4:**
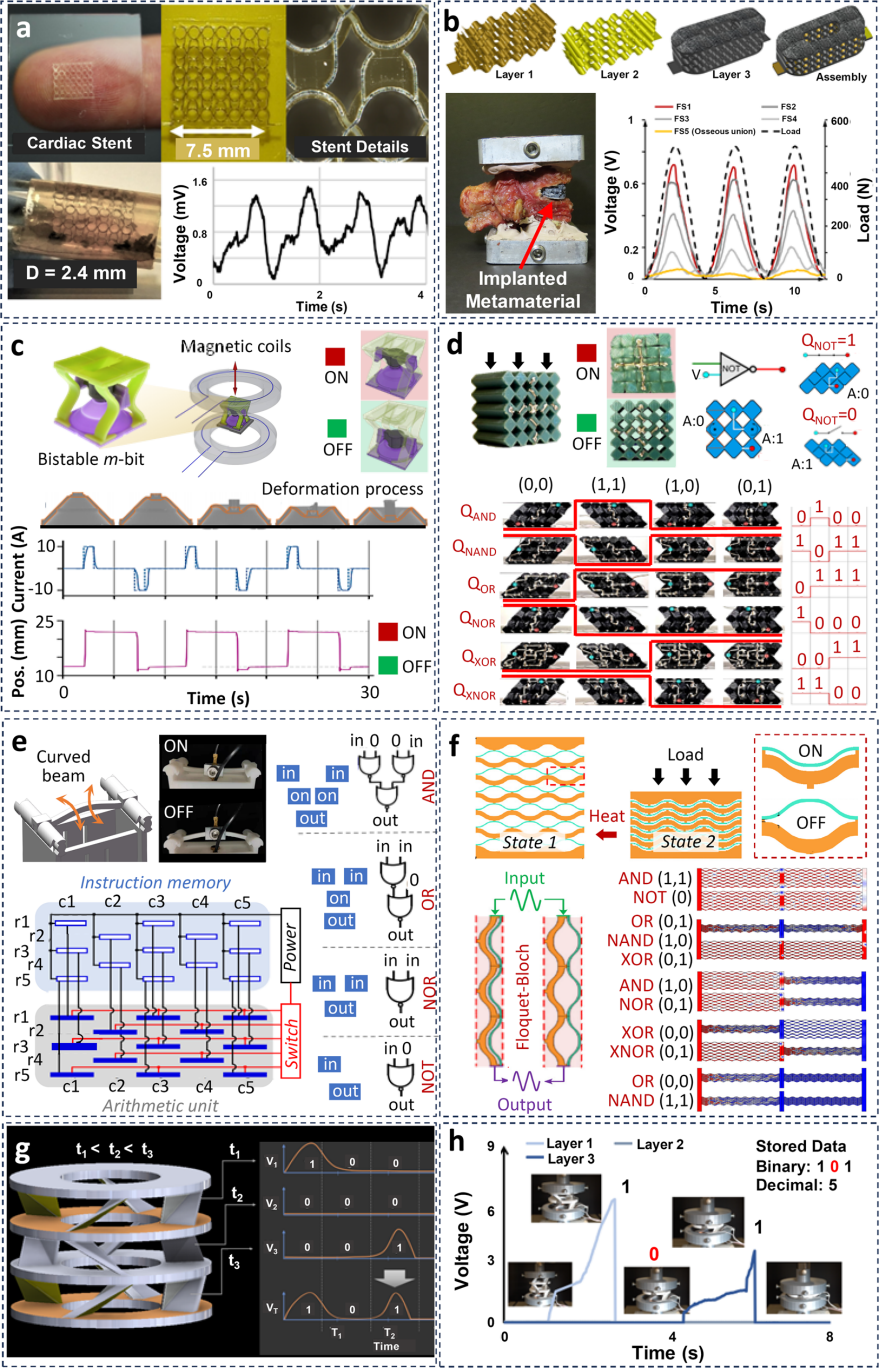
Emerging mechanical metamaterial devices. Examples of multifunctional metamaterial implantable devices with tunable mechanical properties. These implants are capable of self-powering, automatically responding to their environments, and monitoring their condition: (**a**) A metamaterial cardiovascular stent for continuous measurement of the artery radial pressure changes due to tissue overgrowth^12^. **b** A self-powered metamaterial spinal fusion interbody device for monitoring bone healing progress^6^. **c** Tileable mechanical metamaterial with stable memory at the unit-cell level^128^. **d** Cellular mechanical metamaterials composed of conductive polymers to realize all digital logic gates and gate assemblies^23^. **e** Mechanological metamaterials to obtain logical computing by imposing sequential excitations^129^. **f** Elastic mechanical metamaterials with multistable states during the active regulation to adjust the starting and ending frequencies and broaden the frequency ranges of bandgaps and control the elastic wave propagation^22^. **g** Working mechanism of the mechano-responsive data storage metamaterials^160^. **h** An example that shows processing a string of codes “101” and decimal “5” incorporated into the structure of the mechano-responsive metamaterials^160^.

Emerging mechanical metamaterials have begun to enter the era of devices. Mechanical metamaterials have been assembled into larger, integrated networks to form devices capable of completing more complex operations. Mechanical metamaterial devices are mainly functionalized using one or more of three strategies, including (1) merging different mechanical metamaterial components into overall devices to meet certain requirements, (2) combining mechanical metamaterial components with other structures to enhance functionality, and (3) integrating with microprocessors to trigger or control other components, as shown in Fig. 4a. Mechanical metamaterial devices have debuted in active sensing and energy harvesting (e.g^6,12^.). Mechanical metamaterials have been used as components and/or alternatives to combine with other structures and/or replace the parts made of traditional materials. For example, mechanical metamaterial gears fabricated by metal 3D printing have been used to replace certain parts in vehicles as the replaceable components with rapid fabrication period and low cost^159^. Third, mechanical metamaterials have been used as mechanical triggers for microprocessors in various electronic systems. Sensitively stimulated by the external environment, mechanical metamaterials have been used as controllable terminals to trigger the chips for multifunctional applications, such as advanced sensing^12^ or logic operation^22^, data processing^20^, etc. In general, the challenges associated with these applications cannot be easily resolved by traditional design strategies at the structural level (see Fig. 2). Instead, it is necessary to treat mechanical metamaterials as components, and integrate them with other functional parts to achieve the entire multifunctional system.

### Mechanical metamaterial devices with a level of artificial cognition

Electronic devices have dominated digital computation and information processing ever since their debut, mainly due to their superior potential for miniaturization and integration. Enabling mechanical metamaterials with sensing, energy harvesting, digital computing and information storage functionalities is a critical step toward achieving electronic mechanical metamaterials with a level of cognition and primitive intelligence. Cognition is the process of acquiring and using information, while intelligence is a broader concept encompassing a wider range of cognitive abilities. By integrating cognitive abilities into the mechanical metamaterials’ fabric, it is possible to equip them with intelligence, even at the most primitive levels, through which they can acquire knowledge, process it, communicate it, and thus create a sense–decide–respond loop^160^. Such intelligent metastructures can utilize their entire constituent components for self-powering, self-diagnostics, self-repair, self-degradation, digital computing functionality, etc.

To enable mechanical devices with informative processing and data storage in the information era, significant paradigmatic changes have happened in these devices from the conventionally passive to innovatively autonomous^18,22,23,129^. Autonomy in these passive devices is realized by integrating with active sensing and feedback mechanisms, and therefore, autonomous systems are significantly dependent on intelligent matter with the multifunctionalities of actuation, adaptation, and information processing^22,23^. Taking advantage of the response beyond mechanical (e.g., electromechanical or magneto-mechanical characteristics), mechanical metamaterials have been expanded to be a novel type of mechanical logics^129^. The functionalities of mechanical metamaterials in digital computing and information storage are realized at the structural level, embedding mechanical logic in mechanical metamaterials to produce local morphological computation^17,161^. Mechanical informative and computing systems have the potential to complement traditional electronic computing system by tackling some of their limitations such as unstable performance in extreme environments. Given the multidisciplinary nature of the mechanical devices with informative and scientific functions, researchers in mechanical metamaterials will need to be involved with insights and contributions from other fields such as materials science, computer science, information theory, microelectronics, advanced manufacturing, etc.

Emerging mechanical metamaterial devices (especially as singular entities) also exhibit potential advantages in robotic applications. Current robotic systems often require integrating various traditional electronic components into one system leading to challenges in maintaining functionality, especially under harsh conditions. Examples are robotic operations in extreme environments such as deep sea or deep space, applications with specific purposes such as high pressure/high radiation in power plants, or applications where bully electronics cannot be deployed (e.g., medical implants). It is desirable to minimize the complexity associated with integrating numerous electronic pieces into robotic systems, and to move beyond form factor limitations imposed by traditional electronics. Some decision-making capabilities of robots (e.g., trajectory control in response to environmental cues^142^) may be able to be embodied as metamaterial-based multifunctional devices, to increase reliability, robustness, and maintainability. However, this emerging direction is still in its infancy, and it remains to be fully explored. Figure 4a and b display a new generation of multifunctional metamaterial implantable devices capable of automatically responding to their environments and monitoring their conditions^6,12^. The limited studies in this area reveal how miniaturized cardiovascular stents (Fig. 4a) can be used to monitor the artery radial pressure changes due to tissue overgrowth without relying on external electronics^12^, or self-powered mechanical metamaterial spinal fusion constructs (Fig. 4b) can monitor healing progress^6^.

Mechanical logic can be functionalized to enhance traditional robotics controls, but the lack of digital electrical output is a severe limitation of the current embedded mechanical logic systems^17^. Recent studies have demonstrated metamaterial switches designed with conductive ink patterns to convert programmable mechanical deformation into reconfigurable electrical circuits^23^. The mechanical switches encoded different mechanical states into ones “1” and zeros “0” based on their electrical signals. Consequently, mechanical metamaterials with active response are necessary to achieve intelligent devices with self-powered information processing functionalities. Figure 4c presents the design framework for the tileable mechanical metamaterial with stable memory at the unit-cell level^128^. The mechanical metamaterials were designed with an array of physical binary elements (i.e., m-bits that can independently and reversibly switch between two stable states under magnetic actuation), analogous to digital bits, with clearly delineated writing and reading phases. Figure 4d displays the cellular mechanical metamaterials composed of conductive polymers to realize all digital logic gates and gate assemblies^23^. The authors used conductive polymer networks in the metamaterial constituents and correlated mechanical buckling modes with network connectivity to realize conventional logic gates in the soft, conductive matter. Figure 4e presents the mechanological metamaterials to obtain logical computing by imposing sequential excitations^129^. The authors reprogrammed the metamaterials via selectively imposing and releasing the excitations and realized the universal combinatorial logic and sequential logic (memory). The reported mechanological metamaterials can serve as a platform for constructing reusable and multifunctional mechanical systems with strong computation and information processing capability. Figure 4f reports the elastic mechanical metamaterials with multistable states during the active regulation to adjust the starting and ending frequencies and broaden the frequency ranges of bandgaps and control the elastic wave propagation^22^. The authors implemented a bi-stable logic-gate elastic metamaterial to correctly execute simple wave logic operations. Figure 4g and h present a new class of mechanical metamaterials with self-powered digital information storage capability^160^. In the so-called mechanically-responsive data storage metamaterials, the authors incorporated the data into a set of self-recovering unit cells that form the material lattice. These self-powered data storage materials can potentially be used to tackle problems related to developing low-cost, non-volatile, and long-term storage solutions^160,162^. Table 4 summarizes the existing mechanical metamaterial devices for informative and computing applications along with their key advantages.

**Table 4:** A summary of reported mechanical metamaterial devices for applications related to information processing, along with their key advantages

		Category	Characteristics	Potential applications	Advantages	Ref.
Logic gates	Resonant clock network	Wave-based	Designed with an infinite-wavelength zeroth-order resonance mode and utilizes the ultralow Joule loss of superconductors at microwave frequencies	Scaling the power distribution network in superconductor digital circuits to CMOS levels of integration	Metamaterial resonant clock network for energy-efficient power delivery to large superconducting digital systems	^208^
	Terahertz (THz) metamaterials		Programmable THz metamaterials with cut-wire resonator (CWR) sandwiched two face-to-face split-ring resonators.	Stable polarization switch	Coding digits can be switched by changing the vertical distance of the CWR	^209^
	Terahertz (THz) metamaterials		MEMS-based metadevices based on switchable winding-shaped cantilever metamaterial for active logical modulation	Enlarging the operating frequency range, which provides various possibilities in multifunctional switching, active logical modulating, and optical computing applications	Better optical switching performance, realizing a high-efficient optical switch and programmable devices	^210^
	Boolean mechanical logic	Mechanical-based	Performing Boolean logic operation based on the buckling response of 3D unit cells	Complementing the semiconductor electronics for operation in harsh environments (e.g., high radiation fields in nuclear reactors and hot cell laboratories)	Mechanical logic devices to perform various functions (e.g., Boolean logic, sensing or actuating)	^23,160,211^
	Surface plasmon polaritons	Mechanical-based	Coding and programmable designer plasmon polaritons by an ultrathin corrugated metallic strip loaded with active devices and a digital system	Switching polaritons in real time using a single prototype and the digital control system	Digital-analog functions of logical gates based on 1-bit coding, digital phase shifters based on 2-bit coding, and slow waves based on 4-bit coding	^212^
Computing	Mathematical operations	Wave-based	Metamaterial blocks to perform mathematical operations by propagating an impinging wave through these blocks	Direct, ultrafast, wave-based analog computation, equation solving, and signal processing at the hardware level	Wave-based computing systems significantly thinner than conventional lens-based optical signal and data processors	^213^
Image processing	Computational imaging		Low-profile aperture for microwave imaging without lenses, moving parts or phase shifters	Combing computational imaging approach with custom aperture hardware to perform compression in the physical layer	Extending the microwave and millimeter-wave imaging capabilities by the small form factor and lack of moving parts	^214^
Data processing	Data memory	Electromagnetic-based	Tileable mechanical metamaterial with stable memory at the unit-cell level by arraying physical binary elements (m-bits) with clearly delineated writing and reading phases	Stable memory and on-demand programmability of mechanical properties	Distinctly different mechanical response that is fully elastic and can be reversibly cycled	^128^

### Outlook and roadmap

**Fig. 5:**
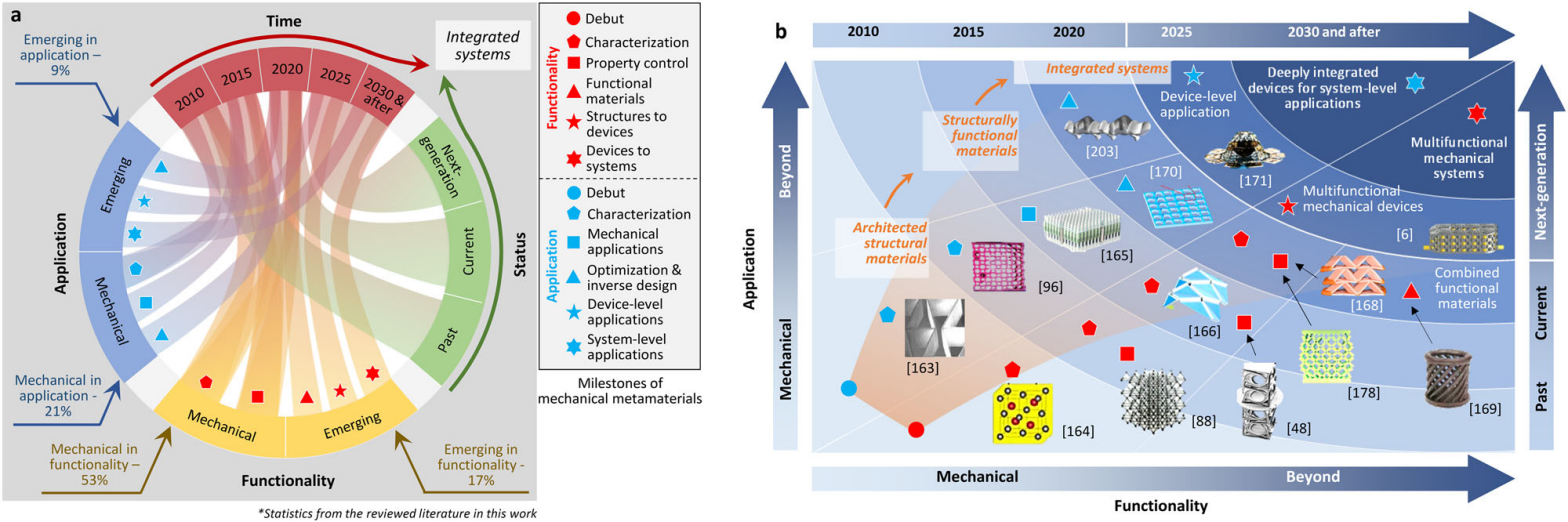
Roadmap toward next-generation intelligent mechanical metamaterial devices and systems. **a** Development context of mechanical metamaterials within and beyond the mechanical domain in terms of the key milestones since 2010. **b** Functionality and application of mechanical metamaterials within and beyond mechanical properties at different stages^6, 48, 88, 96, 157, 163–166, 168–171, 203^.

After nearly two decades of rapid development, mechanical metamaterials have transformed our understanding of advanced functionalities that can be integrated into mechanical materials and structures. Next-generation mechanical metamaterials may possess a degree of intelligence, i.e., the ability to autonomously acquire and process information and act purposefully, while naturally inheriting all unprecedented/counterintuitive mechanical features of classical mechanical metamaterials. These intelligent mechanical metamaterials can be designed as integrated devices and systems to satisfy the requirements in the directions of generalized functionality and specific application, as shown in Fig. 5. Figure 5a illustrates the development context of mechanical metamaterials within and beyond the mechanical domain in terms of the key milestones since 2010. Figure 5b further compares the development of mechanical metamaterials at different stages. Based on the literature reviewed in this work, the studies of mechanical metamaterials during the last two decades are mainly focused on fundamentals (i.e., ~70%), with the remainder being more application-oriented (30%). Different functionalities and potential applications of mechanical metamaterials have been reported within and beyond the mechanical domain at different stages, including the architected structural materials prior to 2010^163,164^, the structurally functional materials in the 2010s^48,88,96,165–167^, the integrated systems with property control^168^ and combination^169^, inverse design^157,170^ and multifunctional devices^6,12,171^ in the 2020 s, and the deeply integrated devices in the 2030 s and after.

The next-generation mechanical metamaterials are approaching integrated systems. More studies are expected to be conducted with the goal of further optimizing their functionalities and expanding the application domains. To this end, current studies of mechanical metamaterials have been conducted in the following three directions. First, materials science has become more and more dominant in the direction of structural materials. In particular, functional materials have been deployed to obtain mechanical metamaterials that are active, adaptable, and capable of actuation. For example, electromechanical, thermomechanical, magneto-mechanical, or optomechanical materials have been used in mechanical metamaterials to achieve tunable, self-adapted responses actuated by external stimuli. Second, data-driven methods have begun to play a key role for researchers exploring the design space of mechanical metamaterials, from the analysis of mechanisms to performance optimization. Third, mechanical metamaterials have begun to be considered as integratable components in devices with controllable functions and performance.

Expansion of mechanical metamaterials beyond mechanical does not exclude other types of metamaterials. Instead, multifunctional applications (e.g., sensing, energy harvesting and communicating) typically require the technical solutions combined by different types of metamaterials in the interdisciplinary fields. Integrating different types of metamaterial components to a system to achieve the functionalities that are impossible for any single type of metamaterial could become an emerging direction to the metamaterials family. Although many recent studies have demonstrated the potential for mechanical metamaterials with functionalities beyond mechanical, these three directions are still facing theoretical and technological challenges. Integrating functional materials has severely increased the difficulty of manufacturing mechanical metamaterials, especially for multiscale fabrication with well reliability and feasibility. AI-enhanced mechanical metamaterials are still at an early stage that mainly emphasizes mechanical properties. Therefore, expanding AI applications to improve the characteristics beyond the mechanical domain will open a bright development avenue for mechanical metamaterials. Deep integration is of significance to fully functionalize mechanical metamaterial devices with high effectiveness. However, this critically relies on the cooperation of the metamaterial components. It is still challenging to maintain the full functionality of the components due to issues of robustness and fabrication imperfections, let alone combining them into one piece for high-effective operation. Further research in these important areas will accelerate the functionality and utility of mechanical metamaterials for various real-life applications.
